# Second Language Learners’ Cognitive Evaluation of GenAI Interactive Learning Experience: Scale Development and Network Analysis

**DOI:** 10.3390/jintelligence14090226

**Published:** 2026-09-19

**Authors:** Hanwei Wu, Gurpinder Singh Lalli, Yongliang Wang

**Affiliations:** 1 Department of Foreign Languages, Basic Education College, National University of Defense Technology, Changsha 410072, China; whf0319@zufe.edu.cn; 2 School of Education, University of Wolverhampton, Wolverhampton WS1 SBD, UK; glalli@wlv.ac.uk; 3 School of Foreign Studies, North China University of Water Resources and Electric Power, Zhengzhou 450046, China

**Keywords:** GenAI, L2 learning, cognitive evaluation, interactive learning experience, scale development, network analysis

## Abstract

As generative artificial intelligence (GenAI) becomes increasingly integrated into second language (L2) learning, there is a growing need for reliable instruments to assess learners’ cognitive evaluation of GenAI-mediated interactive learning experiences. This study developed and validated the GenAI L2 Interactive Learning Experience Scale (AI-L2-ILES) among 1130 Chinese university students. Drawing on an interactive experience perspective and adapting it to L2 learning, the study conceptualized learners’ cognitive evaluation of GenAI interaction as comprising five dimensions: Effectiveness, Sociability, Novelty, Flow, and Seamlessness. Exploratory and confirmatory factor analyses supported this five-factor structure, with satisfactory evidence of reliability, validity, and measurement invariance across gender and academic disciplines. Network analysis further revealed that Flow was the most central dimension, Sociability showed the strongest association with Effectiveness, and Novelty occupied a relatively peripheral position. The network structure was stable across academic disciplines. Overall, the AI-L2-ILES provides a psychometrically sound measure of learners’ cognitive evaluation of GenAI-mediated interactive L2 learning experiences and captures five distinct but interconnected dimensions of this emerging construct. The scale offers a useful tool for examining how L2 learners cognitively evaluate their interactions with GenAI.

## 1. Introduction

In recent years, the application of generative artificial intelligence (GenAI) in second language (L2) education has expanded rapidly. Large language models, represented by ChatGPT, have evolved beyond providing linguistic resources to facilitating learning interactions through their advanced natural language capabilities (Lee et al., 2026). L2 learning is inherently an interactive process, through which learners develop their language abilities via communication, feedback, and negotiation of meaning (Y. Wang & Guo, 2026). Traditionally, interactions in L2 learning have primarily occurred between learners and teachers or peers. However, the integration of GenAI has transformed both interaction partners and processes, enabling learners to engage in continuous, immediate, and personalized language communication with artificial intelligence systems (H. Wu et al., 2026). Therefore, how L2 learners evaluate this emerging human–GenAI interaction process is of great importance.

However, reliable instruments specifically designed to assess L2 learners’ cognitive evaluation of their interactive learning experiences with GenAI remain scarce. Existing scales have mainly focused on learners’ L2 skills developed through GenAI feedback (L. Xu et al., 2025; Yao et al., 2026) or L2 teachers’ GenAI-related literacies (Y. Wang et al., 2025), overlooking learners’ holistic evaluations developed through sustained GenAI interactions. Smart Service Interactive Experience (SSIE) from service psychology provides a relevant framework for understanding such evaluations (Zhang et al., 2025). Although recent studies have extended SSIE to GenAI-assisted education (P. Li, 2026), its original dimensions were developed for service experiences and user satisfaction rather than L2 learning contexts. Therefore, drawing on SSIE and adapting it to L2 learning characteristics, this study develops the GenAI L2 Interactive Learning Experience Scale (AI-L2-ILES) to assess L2 learners’ interactive learning experiences with GenAI.

This study further incorporates network analysis as a complementary approach. While factor analysis examines dimensional structures by identifying which dimensions constitute a construct, it mainly yields zero-order marginal correlations among latent factors. These bivariate associations cannot disentangle genuine direct links from indirect, spurious covariation driven by other dimensions within the construct system (Borsboom et al., 2021); thus, factor-analytic outputs offer limited insights into how dimensions directly interconnect with one another. Conceptualizing constructs as networks of nodes and edges, network analysis estimates partial conditional associations: edge weights reflect pairwise relationships after statistically controlling for all remaining dimensions in the network. This method enables visualization of direct associations, identification of central hub or peripheral dimensions, and mapping of unique pathways among experiential facets that cannot be differentiated from conventional factor-correlation matrices (Borsboom et al., 2021). Previous scale research has applied network analysis following factor validation and demonstrated its complementary value (Niu et al., 2024). Together, factor and network analyses yield a more comprehensive understanding of construct structures. Moreover, although previous research has identified disciplinary differences in L2 learning and GenAI usage needs (Xie, 2019; Y. Xu & Wu, 2026), whether such differences extend to interactive-experience structures remains unclear. Therefore, this study conducts cross-disciplinary network comparisons to examine the stability of the AI-L2-ILES network structure.

Overall, this study aims to develop and validate the AI-L2-ILES while further exploring the structural relationships among its dimensions. Specifically, this study first employs exploratory factor analysis (EFA) and confirmatory factor analysis (CFA) to establish the scale’s factor structure, assess its reliability and validity, and examine measurement invariance across learners from different genders and academic disciplines. Building on these psychometric evaluations, this study further conducts dimension-level network analysis to identify patterns of interrelationships among dimensions and determine their relative importance within the construct. By integrating scale development with network analysis, this study aims to provide a reliable and valid instrument for assessing GenAI L2 interactive learning experience and offer insights into the internal structure of this construct.

## 2. Literature Review

### 2.1. GenAI Interactive Learning Experience as an Experiential Construct

The concept of SSIE is primarily grounded in theories in service-quality management and human–computer interaction. In the service management literature, Parasuraman et al. (1985) proposed the service-quality model, emphasizing that interactive experiences during service delivery constitute a critical factor influencing users’ evaluations of service quality. With the advancement of intelligent technologies, this perspective has been extended to smart-service contexts, where users’ evaluations of intelligent systems are shaped not only by functional performance but also by their overall perceptions of the interaction process (Ashfaq et al., 2025). Meanwhile, the theories of human–computer interaction offer a valuable perspective for understanding user relationships with intelligent systems. Reeves and Nass (1996) demonstrated that individuals naturally apply social norms and expectations to technological systems when interacting with media such as computers. Building on this perspective, Nass and Moon (2000) proposed the Computers Are Social Actors paradigm, highlighting that human–computer interaction involves inherently social processes rather than merely functional exchanges.

The emergence of smart services has prompted researchers to integrate these two theoretical traditions and systematically investigate users’ holistic experiences during interactions with intelligent systems. Prior studies have demonstrated that SSIE is a multidimensional construct. For instance, based on the experiences of hotel guests, Ali et al. (2026) conceptualized SSIE as a five-dimensional construct comprising Seamlessness, Accuracy, Enjoyment, Security, and Empowerment. Similarly, Zhang et al. (2025) developed an SSIE scale for family travelers, identifying eight dimensions: *Efficiency*, *Ease of Use*, *Learning*, *Socialization*, *Novelty*, *Safety, Flow*, and *Seamlessness*. Extending this line of research, Ashfaq et al. (2025) treated the eight-dimensional SSIE as a set of cognitive factors and examined its influence on users’ emotional experiences with hotel service robots.

Based on the aforementioned theoretical foundations, this study defines GenAI L2 interactive learning experience as a comprehensive cognitive evaluation developed by L2 learners through their perceptions of the interaction process, perceived relationship with GenAI, and learning value during sustained interactions with GenAI. This construct highlights that learners do not evaluate GenAI solely based on its functional capabilities or technological performance; rather, they form holistic judgments within specific L2 learning contexts regarding whether learner–GenAI interactions can support learning goals, facilitate meaningful communication, and promote positive interactive experiences. Thus, GenAI L2 interactive learning experience not only reflects the extension of intelligent system interaction characteristics into educational settings but also captures learners’ cognitive construction of GenAI as a meaningful interactive learning partner. This construct focuses on learners’ evaluative appraisals of the interaction process, rather than on the specific cognitive or affective mechanisms that occur within L2 interactions.

To clarify the conceptual boundaries of AI-L2-ILE, it is useful to recognize that the construct is not limited to purely cognitive evaluations, but represents learners’ multidimensional experiential appraisal of AI-mediated L2 interaction. The dimensions of effectiveness, sociability, novelty, flow, and seamlessness capture complementary aspects of how learners evaluate and experience the interaction process. Unlike perceived usefulness and technology acceptance (Davis, 1989), which focus on instrumental evaluations of technology attributes and adoption intentions, AI-L2-ILE captures learners’ holistic perceptions of the interaction process rather than their likelihood of adoption. Unlike usability, which emphasizes ease, efficiency, and effort in system use, AI-L2-ILE encompasses broader experiential facets of interaction. Unlike flow (Csikszentmihalyi, 1990), which primarily refers to an immersive psychological state, AI-L2-ILE encompasses multiple facets of interaction appraisal beyond absorption. Unlike engagement, which emphasizes behavioral or emotional involvement during a task (Eerdemutu et al., 2024), AI-L2-ILE focuses on learners’ appraisal of the interaction experience. Unlike perceived interactivity (F. Wang et al., 2025), which centers on perceived system responsiveness and user control, AI-L2-ILE has a broader scope, encompassing multiple facets of the interaction experience beyond responsiveness. Unlike satisfaction (Deci & Ryan, 2000), which reflects a global evaluation of an experience or outcome, AI-L2-ILE represents a multidimensional appraisal of the interaction experience. In short, AI-L2-ILE is a multidimensional experiential appraisal construct that captures learners’ overall appraisal of the human–GenAI interaction experience.

This construct is not assumed to be entirely context-free. Recent research has highlighted that learners’ interaction experiences with GenAI may vary as a function of task structure. Cosi Mireia and Poole (2025) found that task type influenced learners’ language output more than ChatGPT’s prompted proficiency level. Y. Wang (2026) observed that learners’ engagement with GenAI ranged from deep evaluative use to surface-level dependence depending on task design. Sim et al. (2025) similarly found that ChatGPT’s effectiveness varied across different pragmatic tasks. These findings suggest that learners may experience GenAI differently depending on the task at hand. This clarifies that the scale captures experiential appraisals within specific task contexts.

### 2.2. GenAI-Assisted L2 Learning as the Target Context

The application of GenAI in L2 learning has expanded beyond the provision of linguistic resources to supporting interactive learning processes. Previous research has demonstrated that GenAI can not only provide language feedback and learning support but also function as an interactive partner in language learning. Lee et al. (2026) identified multiple roles of GenAI in language classrooms, including Feedback Provider, Learning Tutor, Cognitive Stimulator, Interaction Facilitator, and Conversation Partner. These roles suggest that GenAI is transitioning from a supplementary tool to an active participant in language practice and learning interactions, underscoring the importance of interactive learning experiences in GenAI-assisted L2 learning. L2 learning is inherently an interactive process, through which learners develop language abilities through communication, feedback, and negotiation of meaning (H. Wu et al., 2026). Traditionally, such interactions have primarily occurred between learners and human interlocutors, such as teachers and peers. However, GenAI introduces a new form of interaction by enabling learners to engage in continuous, immediate, and personalized communication with AI systems. (Pan et al., 2025; C. Wang et al., 2024). Accordingly, how learners perceive and evaluate these emerging human–GenAI interaction experiences may shape their willingness to use GenAI and language learning behaviors. Despite growing research interest in GenAI-assisted L2 learning, existing quantitative studies have primarily focused on functional effectiveness or user attitudes (Saarela et al., 2026), while learners’ holistic experiences during sustained GenAI-mediated interactions remain insufficiently explored. Therefore, developing a theoretically grounded and empirically validated measure of AI-L2-ILE is necessary.

Hitherto, the Perceived Learner–AI Interactivity Scale developed by F. Wang et al. (2025) is the only existing instrument that shows conceptual relevance to this study. However, this scale does not directly assess the construct of GenAI-assisted learning experience. Comprising four dimensions: Responsiveness, Personalization, Learner Control, and Learning Engagement, the scale primarily captures learners’ perceptions of GenAI systems’ interactive capabilities and interactional features. Specifically, it focuses on whether GenAI systems can facilitate effective interaction and emphasizes learners’ cognitive evaluation of interactional functions and quality. In contrast, drawing on the concept of interactive experience in service psychology, the AI-L2-ILE examined in this study represents a broader experiential construct. It focuses on learners’ holistic evaluations of the interaction process, their relationship with GenAI, and the learning value they derive from sustained human–GenAI interactions. Thus, perceived interactivity primarily reflects learners’ perceptions of GenAI’s interactional capabilities and system attributes, whereas interactive learning experience captures how learners interpret and evaluate the meaning and value of GenAI-mediated interactions within L2 learning contexts. Accordingly, the two constructs differ in their conceptual focus and analytical levels.

Recent research has begun to characterize GenAI-supported L2 learning along several pedagogical dimensions. First, GenAI can engage learners in task-based language practice, where learners complete meaningful communicative tasks (e.g., drafting emails, role-playing conversations, composing essays) with GenAI as a conversational partner or co-writer (Y. Li et al., 2026). Second, GenAI facilitates real-time feedback and meaning negotiation, providing immediate corrective or reformulative responses that allow learners to notice gaps and modify their output (Pan et al., 2025). Third, beyond classroom settings, GenAI enables self-directed and informal L2 practice, as learners independently use GenAI tools for language exploration, exposure, and production outside formal instruction (G. L. Liu & Zhao, 2026). These pedagogical features, such as task engagement, feedback exchange, and autonomous practice, distinguish GenAI-mediated L2 interaction from general smart service encounters and underscore the need for a context-sensitive measure that captures learners’ experiential evaluations in this specific educational setting.

### 2.3. Rationales for Contextualizing SSIE in GenAI-Assisted L2 Learning

This study adopts a contextual adaptation approach (DeVellis, 2016), drawing on the SSIE scale developed by Zhang et al. (2025). Specifically, the SSIE dimensional framework is adapted and refined to develop a measure of AI-L2-ILE. The selection of SSIE as the theoretical basis is motivated by its conceptual relevance and potential for contextual extension. As a relatively recent and well-established framework in the field of smart service research, SSIE comprises eight dimensions that capture key experiential characteristics underlying users’ interactions with intelligent systems (Zhang et al., 2025). GenAI, as an emerging form of intelligent service technology, shares fundamental similarities with smart services, as both involve continuous, dynamic, and bidirectional interactions between users and intelligent systems. F. Wang et al. (2025) further suggested that learners’ perceptions developed through GenAI interactions share psychological continuity with experiences involving other intelligent systems. Therefore, SSIE offers a valuable theoretical lens for understanding learners’ interactive experiences with GenAI.

However, SSIE was not originally developed for educational contexts, and its initial application scenarios differ substantially from GenAI-assisted L2 learning. Smart service contexts primarily focus on user experience, service value, and satisfaction, whereas L2 learning contexts emphasize cognitive engagement, interaction quality, and learning support (Pan et al., 2025; C. Wang et al., 2024). Therefore, the meanings of SSIE dimensions require contextual reinterpretation to capture learners’ unique experiences in GenAI-assisted L2 environments. For instance, *Socialization* in GenAI interactions does not necessarily refer to the social connections among users typically emphasized in service contexts; rather, it may reflect whether GenAI can provide partner-like communication, personalized feedback, and learning companionship. Similarly, *Flow* in L2 learning involves not only enjoyment during interaction but also learners’ immersion and sustained engagement while completing learning tasks with GenAI support (H. Wu & Wang, 2025).

Importantly, SSIE is not intended to capture the full range of L2 learning processes. L2 learning involves domain-specific processes such as feedback uptake, negotiation of meaning, self-regulation, and language practice, which are beyond the scope of the present construct (S. Li et al., 2024; Loewen & Sato, 2018; Nassaji, 2016). Rather, SSIE provides an experiential lens for examining how learners holistically evaluate their sustained interactions with GenAI in an L2 learning context. In this sense, L2 learning defines the contextual and functional meaning of the interaction, while SSIE captures learners’ experiential evaluations of that interaction. The AI-L2-ILES therefore measures the experiential layer of GenAI-assisted L2 learning rather than L2 learning processes or outcomes themselves. This distinction also motivates the contextual adaptation of SSIE dimensions to reflect the specific goals and characteristics of L2 learning.

To summarize, SSIE provides a theoretically grounded framework for understanding interactions between users and intelligent systems; however, it was not specifically developed for educational contexts, particularly L2 learning environments. Although recent studies have begun to extend SSIE to GenAI-supported education (e.g., P. Li, 2026 applied the SSIE framework to a GenAI-assisted music education context), empirical research remains limited, and no instrument has been developed specifically to assess learners’ AI-L2-ILE. Therefore, this study builds on the SSIE framework while adapting and reconceptualizing its relevant dimensions in accordance with the characteristics of GenAI-assisted L2 learning. Beyond scale development, this study further employs network analysis as a complementary approach to examine the interrelationships and relative importance of different dimensions (Borsboom et al., 2021), providing additional insights into the internal structure of AI-L2-ILES. Accordingly, this study addresses the following research questions:

**RQ1**: What are the psychometric properties of the AI-L2-ILES?
  ***RQ1a***: What is the underlying factor structure of the AI-L2-ILES?
  ***RQ1b***: Does the AI-L2-ILES demonstrate satisfactory reliability and validity?
  ***RQ1c***: Is the measurement invariance of the AI-L2-ILES supported across different groups?
**RQ2**: How are the dimensions of the AI-L2-ILES interconnected, and what are their relative roles within the construct?

## 3. Methodology

### 3.1. Participants

For sampling, convenience sampling was used. Recruitment announcements were posted on course-management platforms and student WeChat groups of five Chinese universities, inviting students who had used GenAI tools (e.g., ChatGPT, DeepSeek, Kimi) for L2 learning purposes. Inclusion criteria were: (a) current enrollment at one of the five participating universities, and (b) at least a month of experience using GenAI for L2 learning. Prior GenAI-assisted L2 learning experience was determined by two screening questions at the beginning of the questionnaire: one asking whether they had ever used GenAI tools for L2 learning (yes/no), and another asking about the frequency of such use (with options ranging from “rarely” to “daily”). Only those who answered “yes” and reported at least monthly use were included.

Data were collected between 16 and 24 December 2025, from five universities in Hunan, Henan, Jiangsu, and Zhejiang provinces, China. The participating institutions comprised two teacher-training universities, one university of science and technology, one comprehensive university, and one university of finance and economics. Participation was voluntary, and students reported using GenAI tools primarily for L2 learning purposes. No incentives were offered for participation.

Eligible students completed the questionnaire online through Wenjuanxing. Of the 1664 responses initially received, 534 were excluded: 467 failed at least one of the three attention-check items, and 67 contained incomplete data (i.e., missing responses to one or more items). This resulted in 1130 valid responses. The valid sample was then randomly divided into two equal subsamples (*n* = 565 each) for EFA and CFA using SPSS’s random case-selection function. The demographic characteristics of the two subsamples were broadly comparable (see Table 1).

Demographic information, including gender, age, major, and grade, is presented in Table 1. As part of the demographic background, self-rated L2 proficiency was also collected using a single item: “*How would you rate your overall English proficiency?*” with five response options: 1 = very poor, 2 = poor, 3 = average, 4 = good, 5 = very good. In the EFA subsample (*n* = 565), the distribution was: 8 (1.4%) for “*very poor*”, 56 (9.9%) for “*poor*”, 379 (67.1%) for “*average*”, 112 (19.8%) for “*good*”, and 10 (1.8%) for “*very good*”. In the CFA subsample (*n* = 565), the distribution was: 30 (5.3%) for 1 (*very poor)*, 64 (11.3%) for 2 (*poor*), 379 (67.1%) for 3 (*average*), 76 (13.5%) for 4 (*good*), and 16 (2.8%) for 5 (*very good*). The majority of participants in both subsamples rated themselves at or above the midpoint, indicating that most participants perceived themselves as having at least average English proficiency. All of them had received formal English instruction throughout their schooling (from primary to tertiary level), as English is a compulsory subject in China.

### 3.2. Dimensions and Items for AI-L2-ILES

Following the scale-development procedures recommended by DeVellis (2016) and Boateng et al. (2018), the AI-L2-ILES was developed through a contextual adaptation approach. Specifically, the SSIE scale developed by Zhang et al. (2025) served as the theoretical foundation, and its dimensional structure was adapted to capture learners’ interaction experiences with GenAI in L2 learning contexts.

The applicability of the eight SSIE dimensions (i.e., *Efficiency*, *Ease of use*, *Learning*, *Socialization*, *Novelty*, *Safety*, *Flow*, and *Seamlessness*) was examined through expert evaluation and learner discussions. Following McKenzie et al.’s (1999) recommendation, six specialists from semantics (*n* = 2), psychometrics (*n* = 2), and computer-assisted language learning (*n* = 2) evaluated the conceptual relevance and clarity of the adapted dimensions and items. In addition, 27 university students from diverse disciplines (L2, humanities and social sciences, and STEM) and educational levels (undergraduate, master’s, and doctoral; three from each subgroup) assessed the contextual appropriateness of the SSIE dimensions based on their GenAI-assisted L2 learning experiences.

All items were drafted in Chinese and reviewed by six specialists, who provided written comments on item relevance, clarity, and contextual appropriateness. Based on their feedback, the wording was revised to improve clarity and conceptual alignment. The revised items were then discussed with 27 university students in focus-group sessions, where students identified ambiguous phrasing and assessed whether the items accurately reflected their GenAI-assisted L2 learning experiences. Their oral feedback was summarized and used to further refine the wording. Back-translation was not performed because the items were originally developed in Chinese for Chinese-speaking participants. Cognitive interviewing was also not conducted; however, the focus-group discussions served a similar function by identifying ambiguous phrasing and assessing item comprehension. No quantitative content-validity indices were calculated, as expert and learner evaluations were conducted as qualitative content-validation procedures rather than quantitative ratings. This approach was considered appropriate for the initial item-generation and refinement stage (DeVellis, 2016).

Based on these evaluations, *Ease to use* and *Safety* were removed due to their limited relevance for the present sample (Chinese university students with prior GenAI experience). In this context, the GenAI platforms commonly used by the participants (e.g., ChatGPT, DeepSeek, Kimi) feature relatively intuitive interaction mechanisms, and usability was not a differentiating factor in their experience appraisals. In the 27 student discussions, participants reported that their GenAI use in L2 learning primarily involved routine activities such as essay writing, speaking practice, and grammar explanation. Although privacy, inaccurate information, bias, and academic-integrity concerns may arise in GenAI use, these were not identified as salient features of their routine L2 learning interactions. *Safety* was therefore excluded because it primarily concerns risk and responsible use rather than the experiential quality of interaction targeted by the present construct. Similarly, *Ease to use* concerns system usability, whereas “little effort” in the retained items refers to obtaining learning support efficiently. We acknowledge that these dimensions may remain relevant in other settings (e.g., assessment contexts).

*Socialization* was retained but conceptually revised. While the original SSIE dimension emphasizes interpersonal social connections, learners highlighted their perceptions of GenAI as a socially responsive interaction partner during L2 learning. Therefore, this dimension was redefined to capture the social characteristics of GenAI-based learning interactions. Consequently, the adapted initial AI-L2-ILES consisted of six dimensions: *Efficiency*, *Learning*, *Sociability*, *Novelty*, *Flow*, and *Seamlessness*.

The 18 initial items were developed through two approaches. For dimensions with direct conceptual counterparts in the SSIE framework (*Efficiency*, *Socialization*, *Novelty*, *Flow*, and *Seamlessness*), items were adapted from the original SSIE scale (Zhang et al., 2025) by replacing service-domain referents (e.g., “smart technologies”, “hotel stay”) with L2-learning referents (e.g., “GenAI”, “L2 learning”), while preserving the core semantic structure of each item. For the Learning dimension, which had no direct counterpart in SSIE, items were newly developed based on the conceptual definition of learning-related interaction experiences. Table 2 illustrates representative adaptations.

Given the multidimensional nature of the construct, three items were initially developed for each dimension, resulting in an initial pool of 18 items. This item allocation was consistent with Zhang et al.’s (2025) SSIE scale, in which most dimensions were represented by approximately three items. Maintaining a concise scale was intended to balance construct coverage and parsimony, thereby reducing respondent burden and improving response quality. All items are rated on a 6-point Likert scale ranging from 1 (*strongly disagree*) to 6 (*strongly agree*).

### 3.3. GenAI-IDLE as the Criterion

The scale was developed and validated by G. L. Liu et al. (2025) and consists of eight items measuring the frequency and diversity of learners’ use of GenAI for informal English-learning purposes. All items are rated on a 6-point Likert scale ranging from 1 (*strongly disagree*) to 6 (*strongly agree*). In the original scale, “English” was used; for the present study, it was replaced with “L2,” and the items were presented with a parenthetical note specifying that L2 refers to the language being learned (e.g., English), so that the meaning was clear to all participants. The modification was limited to this terminological substitution; the substantive item content and response format remained unchanged. Formal measurement equivalence testing between the original and modified versions was not conducted because the modification was limited to terminology and did not alter the substantive content of the items. Thus, the present analysis provides evidence regarding the internal structure of the modified version, but does not constitute formal evidence of measurement equivalence with the original version. The modified scale demonstrated a unidimensional structure with good model fit: χ^2^/*df* = 2.880, CFI = 0.990, TLI = 0.985, RMSEA = 0.058, SRMR = 0.019. The internal consistency was also high (McDonald’s ω = 0.938).

GenAI-IDLE was selected as the criterion-related measure because it captures a behavioral dimension of learners’ engagement with GenAI that is conceptually related to, yet distinct from, the construct assessed by the AI-L2-ILES. Specifically, the AI-L2-ILES measures learners’ multidimensional appraisal of their interactive learning experiences with GenAI, whereas GenAI-IDLE assesses the frequency and diversity of learners’ autonomous GenAI use for informal L2 learning. Given that positive interactive experiences may foster continued behavioral engagement with technology-mediated systems (Mpinganjira et al., 2025), a positive association between the two constructs is theoretically plausible. Accordingly, a positive correlation between the AI-L2-ILES and GenAI-IDLE was expected to provide preliminary evidence of criterion-related validity, rather than evidence of criterion validity in the strict sense.

More specifically, the characteristics of informal, autonomous, and task-oriented GenAI use provide a theoretical basis for this expected association. Sustained engagement with GenAI across self-selected L2 learning tasks gives learners repeated opportunities to experience and evaluate its affordances, such as responsiveness, continuity, and adaptability. Over time, these accumulated interactional experiences may strengthen perceptions of GenAI’s effectiveness for accomplishing learning goals, while also making interactions feel more seamless and facilitating a greater sense of flow (H. Wu & Wang, 2025). In turn, positive experiences of effectiveness, flow, and seamlessness may encourage learners to continue returning to GenAI for self-directed L2 learning (Y. Xu & Wu, 2026). Thus, the behavioral engagement captured by GenAI-IDLE is theoretically related to the interactional appraisal captured by the AI-L2-ILES, providing a rationale for examining their association. However, because both measures were self-reported and administered concurrently, the observed correlation may also reflect construct overlap and common-method variance. The resulting association should therefore be interpreted as preliminary evidence of criterion-related validity rather than definitive evidence of criterion validity.

### 3.4. Data Analysis

To address **RQ1**, data from Sample 1 were analyzed using R 4.5.3 and the *psych* package. Exploratory factor analysis (EFA) was conducted using maximum likelihood (ML) extraction and oblique Promax rotation, given the expected correlations among factors (Fabrigar et al., 1999; Osborne et al., 2008). Data suitability was assessed using the Kaiser–Meyer–Olkin (KMO) measure and Bartlett’s test of sphericity. Factor retention was informed by multiple criteria, including Kaiser’s criterion (eigenvalues > 1), scree plot inspection, parallel analysis based on 500 simulated datasets, and theoretical interpretability (Hayton et al., 2004). Items were evaluated based on factor loadings and cross-loadings, with loadings above 0.40 considered acceptable. Items showing a difference in less than 0.15 between their two highest loadings were considered for removal. The EFA was rerun after item removal to obtain the final factor solution.

Data from Sample 2 were used for confirmatory factor analysis (CFA) and validation analyses. All CFA models were estimated using AMOS 24.0 with ML estimation. Although the items were measured on a six-point Likert scale, previous simulation research suggests that ML estimation can perform adequately with ordinal indicators having five or more response categories, particularly when category thresholds are approximately symmetric (Rhemtulla et al., 2012). Normality was assessed by examining skewness and kurtosis for each item. Skewness values ranged from –0.517 to 0.382, and kurtosis values ranged from –0.955 to 0.660, both within the acceptable thresholds of |skewness| < 3 and |kurtosis| < 7 (Kline, 2016), indicating no severe violation of normality.

No missing data were present in the final sample. Model fit was evaluated using the chi-square statistic divided by degrees of freedom (χ^2^/*df*), root mean square error of approximation (RMSEA), standardized root mean square residual (SRMR), comparative fit index (CFI), and Tucker–Lewis index (TLI) (Hu & Bentler, 1999). Convergent validity was assessed using average variance extracted (AVE), whereas discriminant validity was assessed using the heterotrait–monotrait ratio (HTMT) and the Fornell–Larcker criterion (Fornell & Larcker, 1981; Henseler et al., 2015). Criterion validity was examined through associations with theoretically relevant external variables. Reliability was evaluated using McDonald’s ω, computed using the OMEGA macro (Hayes & Coutts, 2020) in SPSS 26.0 (Viladrich et al., 2017).

Gender and academic discipline were selected as grouping variables because prior L2 research has identified systematic differences in technology engagement and learning perceptions across these two dimensions. For gender, previous studies have reported gender differences in L2 learners’ perceptions of GenAI tools (Al-atrash et al., 2026; Dolenc & Brumen, 2024). For academic discipline, recent evidence suggests that disciplinary background is associated with differences in students’ use and perceptions of AI-assisted L2 learning (C. Wang et al., 2026; J. Wu et al., 2026). Measurement invariance across gender and academic disciplines was tested using successive model comparisons with ML estimation, where metric invariance constrained factor loadings and scalar invariance constrained both loadings and intercepts across groups. ΔCFI < 0.01 and ΔRMSEA < 0.015 indicated acceptable invariance (Cheung & Rensvold, 2002).

Regarding **RQ2**, network analysis was performed in R (version 4.5.3) using the full sample (*n* = 1130). A Gaussian graphical model was estimated to examine the conditional associations among the five dimensions, with each dimension represented as a node in the network. Node scores were calculated as the mean of items within each dimension (Epskamp et al., 2018). All variables were standardized prior to analysis. Dimension-level analysis was chosen because the five dimensions were empirically confirmed by EFA and CFA, making them appropriate units for examining dimensional interrelationships, and a five-node network provides a parsimonious and interpretable overview of the construct’s higher-order structure. This approach is exploratory and descriptive and does not claim to reveal item-level mechanisms.

The network was estimated using the R packages qgraph (version 1.9.8) and bootnet (version 1.5.7). To obtain a parsimonious network structure, the graphical LASSO with extended Bayesian information criterion (EBIC) tuning was applied, with the hyperparameter γ set to 0.5, the default and recommended value for exploratory network estimation (Epskamp & Fried, 2018). No missing data were present in the full sample after data screening (see Section 3.1), so no imputation was necessary. The accuracy of edge estimates was evaluated through 1000 non-parametric bootstrap iterations, generating 95% confidence intervals for edge weights.

To examine the structural importance of each dimension, centrality indices, including strength and expected influence, were calculated. These indices were used to identify dimensions that played more prominent roles within the network structure (Borsboom et al., 2021). Given the small number of nodes (five dimensions), centrality indices, particularly betweenness and closeness, were not included as these indices are more informative in larger networks (Borsboom et al., 2021).

To examine differences in the AI-L2-ILES network across academic disciplines, pairwise Network Comparison Tests (NCTs) were conducted using the full sample (*n* = 1130) across L2, Humanities and Social Sciences, and STEM groups (van Borkulo et al., 2023). The NCTs used 1000 permutations and examined global strength invariance and network structure invariance. Global strength assessed differences in overall network connectivity, whereas network structure assessed differences in the pattern and magnitude of edge weights. FDR correction was applied to account for pairwise comparisons. Nonsignificant results were interpreted as indicating no statistically detectable between-group differences, rather than identical networks.

In addition, the robustness of the network was examined using case-dropping bootstrap procedures. The correlation stability coefficients (CS) for centrality indices and edge weights were evaluated, with values above 0.50 considered indicative of acceptable stability (Epskamp et al., 2018).

## 4. Results

### 4.1. Scale-Development Findings

#### 4.1.1. Exploratory Factor Analysis

To address ***RQ1a***, EFA was conducted using data from Sample 1. The suitability of the data for factor analysis was initially assessed using KMO measure and Bartlett’s test of sphericity. For the original 18-item scale, the KMO value was 0.896, and Bartlett’s test of sphericity was significant, χ^2^(153) = 6376.488, *p* < 0.001, indicating that the data were suitable for factor analysis. The Likert-type items were treated as approximately continuous, and Pearson correlations were used in the analysis. ML was used as the extraction method, and Promax rotation was applied to allow the factors to correlate.

Initial EFA was conducted on the original 18 items to examine the factor structure and identify potentially problematic items. During the initial EFA, two items from the original *Learning* dimension, I4 and I5, exhibited substantial cross-loadings with *Sociability*. I4 loaded 0.430 on the *Learning* factor and 0.384 on *Sociability*, whereas I5 loaded 0.407 on *Learning* and 0.432 on *Sociability*. The differences between the two factor loadings were 0.046 and 0.025, respectively, both of which are below the predefined threshold of 0.15. These two items were therefore removed because they showed insufficient discrimination between the two dimensions. The EFA was subsequently rerun using the remaining 16 items.

**Figure 1:**
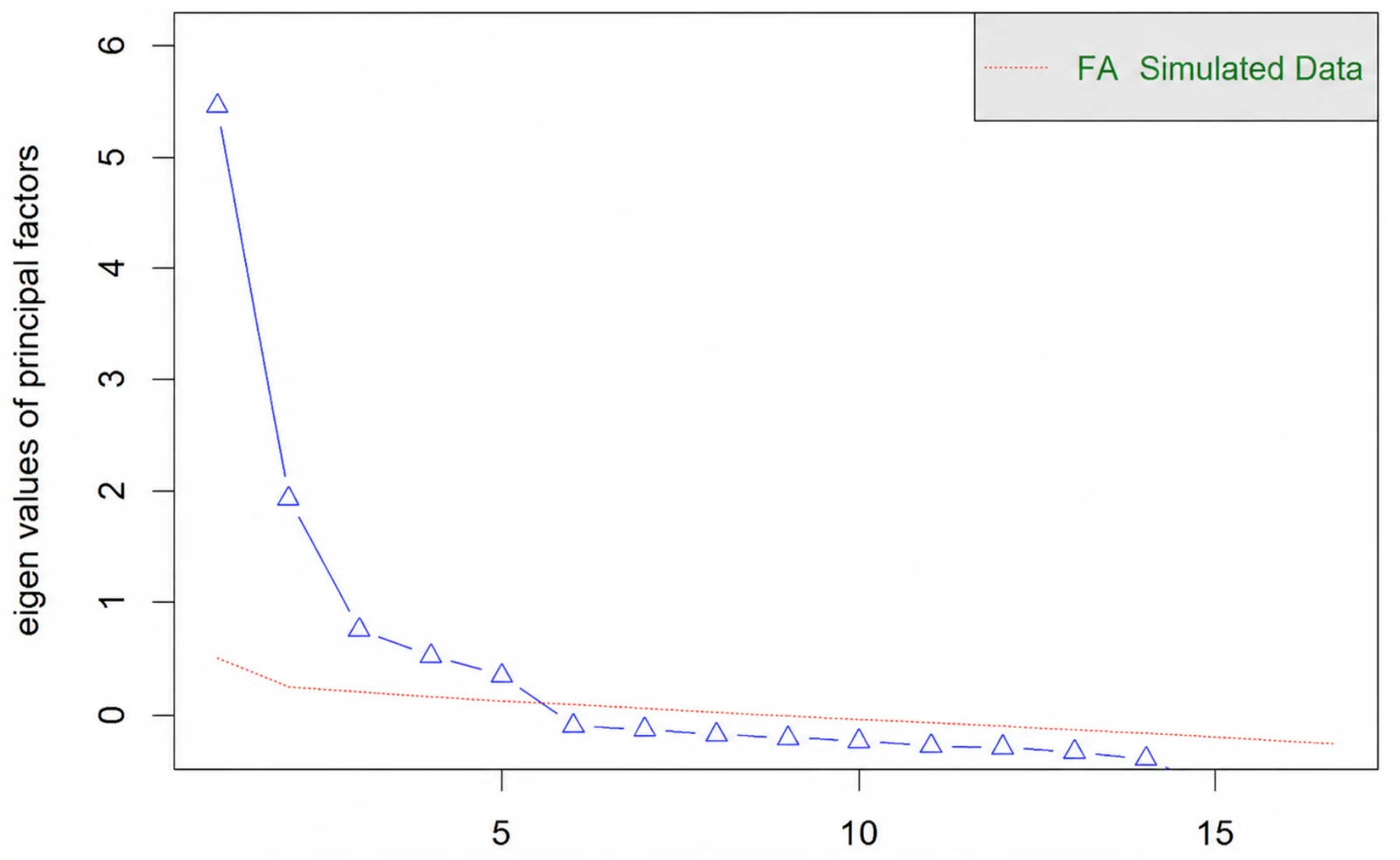
Parallel analysis of the final 16-item scale.

Factor retention was further evaluated using parallel analysis after the item refinement. Parallel analysis of the original 18-item scale suggested six factors; however, the sixth factor was defined primarily by I4 and I5, the two items that exhibited substantial cross-loadings in the initial five-factor solution. Given their insufficient discrimination and the resulting two-item factor, these items were removed before determining the final factor structure. Parallel analysis was then conducted on the remaining 16 items using 500 simulated datasets and maximum likelihood extraction. The results indicated that five factors should be retained (see Figure 1). Accordingly, a five-factor solution was specified for the final EFA.

For the final 16-item solution, the KMO value was 0.873, and Bartlett’s test of sphericity was significant, χ^2^(120) = 5325.410, *p* < 0.001, further supporting the factorability of the data. The final EFA used ML extraction with Promax rotation, consistent with the correlated nature of the factors. The five-factor solution accounted for 67.5% of the total variance. Specifically, *Effectiveness* accounted for 15.6% of the variance, *Sociability* for 14.9%, *Novelty* for 13.0%, *Flow* for 12.0%, and *Seamlessness* for 11.9%.

Notably, the original *Learning* and *Efficiency* dimensions did not emerge as two distinct factors. Instead, one item originally assigned to *Learning* and three items originally assigned to *Efficiency* converged into a single factor. Because these items collectively reflect learners’ perceptions of the effectiveness of GenAI in supporting and facilitating their L2 learning processes, this factor was labeled *Effectiveness*. The label therefore reflects the common substantive meaning of the items while acknowledging the empirical integration of the original *Learning* and *Efficiency* dimensions.

The final EFA solution consisted of five factors: *Effectiveness, Sociability, Novelty, Flow,* and *Seamlessness*. The *Effectiveness* factor comprised four items (I1, I2, I3, and I6), with primary factor loadings ranging from 0.540 to 0.951. *Sociability* comprised three items (I10, I11, and I12), with loadings ranging from 0.776 to 0.991. *Novelty* comprised three items (I7, I8, and I9), with loadings ranging from 0.736 to 0.854. *Flow* comprised three items (I13, I14, and I15), with loadings ranging from 0.690 to 0.838. Finally, *Seamlessness* comprised three items (I16, I17, and I18), with loadings ranging from 0.722 to 0.884. The complete rotated pattern matrix, including primary factor loadings, cross-loadings, and communalities, is presented in Table 3. In addition, the detailed item contents are provided in the Supplementary Materials. The factor correlation matrix is presented in Table 4.

To further clarify the substantive meaning of each retained factor, Table 5 provides a concise interpretation of the five subscales.

#### 4.1.2. Confirmatory Factor Analysis

**Figure 2:**
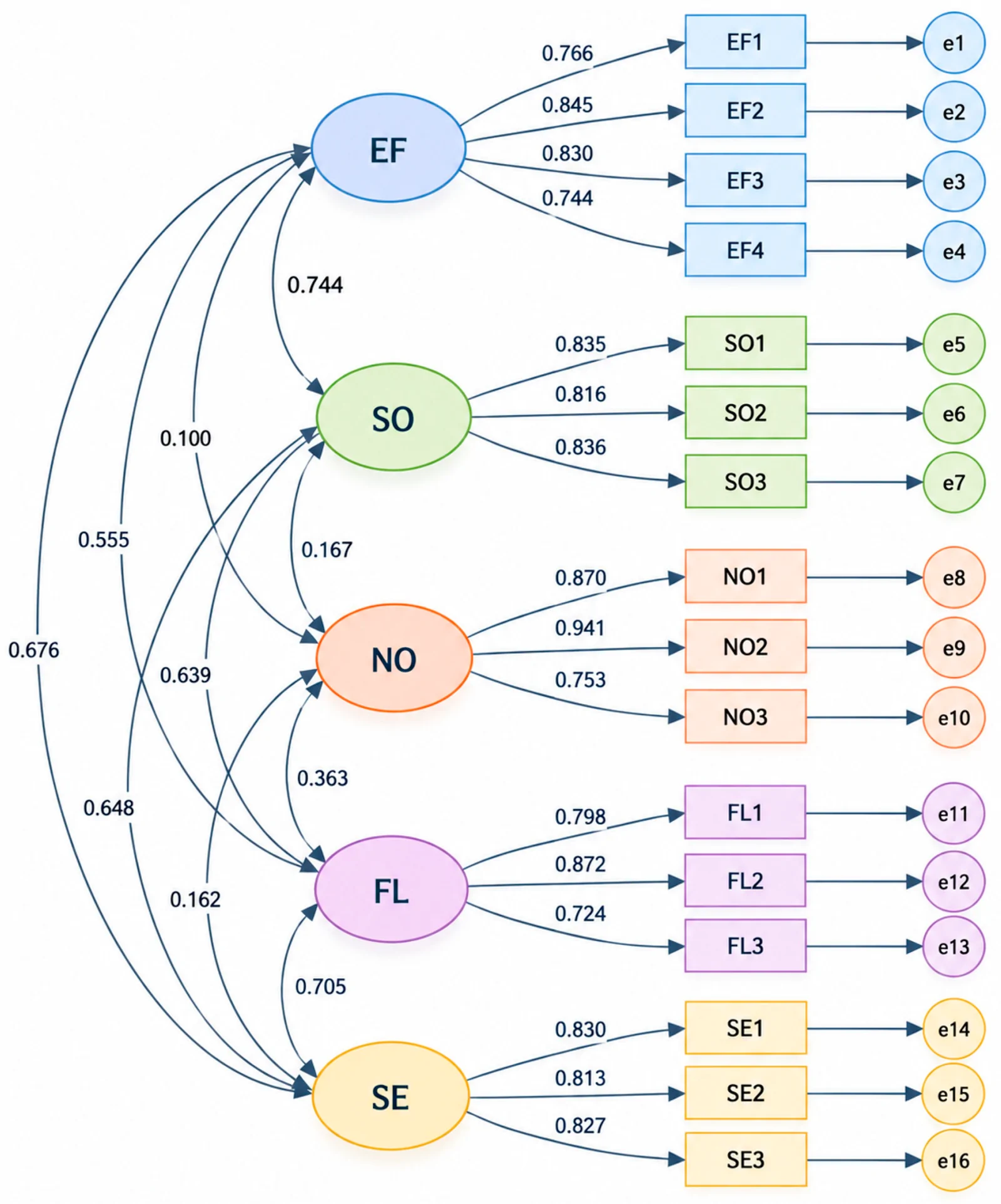
The CFA model of the AI-L2-ILES. *Note*. EF = Effectiveness; SO = Sociability; NO = Novelty; FL = Flow; SE = Seamlessness.

Using the five-factor structure identified in the EFA, the 16-item scale was further examined using data from Sample 2. For ***RQ1b***, CFA was conducted to evaluate whether the proposed factor structure adequately represented the observed data. All items exhibited significant standardized factor loadings, with values exceeding the recommended threshold of 0.45 (see Figure 2, Table 6 and Table 7) (Kline, 2016). Therefore, all 16 items were retained for subsequent analyses.

The model fit indices indicated an acceptable fit without any modifications between the hypothesized five-factor model and the data. Specifically, χ^2^/*df* = 3.062 (<5). Other fit indices demonstrated satisfactory model fit, including TLI = 0.956 and CFI = 0.966 (both > 0.9). In addition, the RMSEA was 0.060 (<0.08), and the SRMR was 0.039 (<0.08) (Hu & Bentler, 1999).

To further validate the five-factor structure, we tested a set of competing models: (a) a one-factor model with all 16 items loading on a single factor; (b) a four-factor model combining Effectiveness and Sociability into a single factor, given their high correlation (*r* = 0.744) in the five-factor solution; and (c) a higher-order model with all five first-order factors loading on a single second-order factor.

The original six-factor model separating *Efficiency* and *Learning* was not estimable because the *Learning* dimension was reduced to a single item after EFA (the other two *Learning* items were removed due to cross-loadings), and a single-item factor is not identified in CFA (Kline, 2016).

As shown in Table 8, the five-factor model demonstrated superior fit compared to the one-factor and four-factor alternatives. The higher-order model also showed acceptable fit, indicating that the five dimensions can be meaningfully summarized by a general construct. These results collectively support the adequacy of the proposed five-factor structure of the AI-L2-ILES.

#### 4.1.3. Validity and Reliability Analysis

To address ***RQ1b***, reliability was assessed using McDonald’s ω, which is conceptually appropriate, as it does not require the tau-equivalence assumption underlying Cronbach’s α. The overall scale demonstrated strong internal consistency (ω = 0.885). For the five subscales, ω values were 0.872 (*Effectiveness*), 0.868 (*Sociability*), 0.889 (*Novelty*), 0.836 (*Flow*), and 0.864 (*Seamlessness*). All values exceeded the recommended threshold of 0.70 (Viladrich et al., 2017), confirming satisfactory reliability of the scale and its dimensions.

Convergent and discriminant validity were then assessed using average variance extracted (AVE) and the Fornell–Larcker criterion. As shown in Table 9, AVE values ranged from 0.635 to 0.737, exceeding the recommended threshold of 0.50 (Fornell & Larcker, 1981), indicating that the items within each factor adequately captured their corresponding constructs.

Discriminant validity was examined using both the Fornell–Larcker criterion and the HTMT ratio of correlations. The HTMT values (see Table 10) for all factor pairs ranged from 0.103 to 0.765, all below the recommended threshold of 0.85 (Henseler et al., 2015), supporting the distinctiveness of the five dimensions. The Fornell–Larcker criterion further supported discriminant validity: the square roots of AVE for all five dimensions (ranging from 0.797 to 0.858) were greater than their correlations with other dimensions (*r* = 0.100–0.705). These results collectively confirm that each dimension is empirically distinct.

In addition, correlation analysis showed that all five AI-L2-ILES subscales were significantly and positively correlated with GenAI-IDLE (all *p*s < 0.001). Specifically, *Effectiveness* was positively correlated with GenAI-IDLE (*r* = 0.579), *Sociability* was positively correlated with GenAI-IDLE (*r* = 0.565), *Novelty* showed a relatively weak positive correlation with GenAI-IDLE (*r* = 0.268), *Flow* was positively correlated with GenAI-IDLE (*r* = 0.625), and *Seamlessness* showed a relatively strong positive correlation with GenAI-IDLE (*r* = 0.670).

These findings provide preliminary evidence for the criterion validity of the scale. However, given that both measures were self-reports collected concurrently from the same participants, the results should be interpreted with caution due to the possibility of common-method variance.

#### 4.1.4. Cross-Group Invariance Analysis

Using data from Sample 2, measurement invariance across gender and academic disciplines was examined to address ***RQ1c*** (see Table 11). For gender, the configural, metric, and scalar invariance models all demonstrated acceptable fit. The changes in fit indices were minimal (ΔCFI < 0.01 and ΔRMSEA < 0.015), supporting scalar invariance across gender groups.

Measurement invariance across academic disciplines was also supported. Although the fit indices slightly decreased across increasingly constrained models, both metric and scalar invariance models met the recommended criteria (ΔCFI < 0.01 and ΔRMSEA < 0.015). These findings indicate that the AI-L2-ILES demonstrates measurement equivalence across genders and academic disciplinary groups.

### 4.2. Network Analysis Findings

#### 4.2.1. Network Structure and Edge Weights

**Figure 3:**
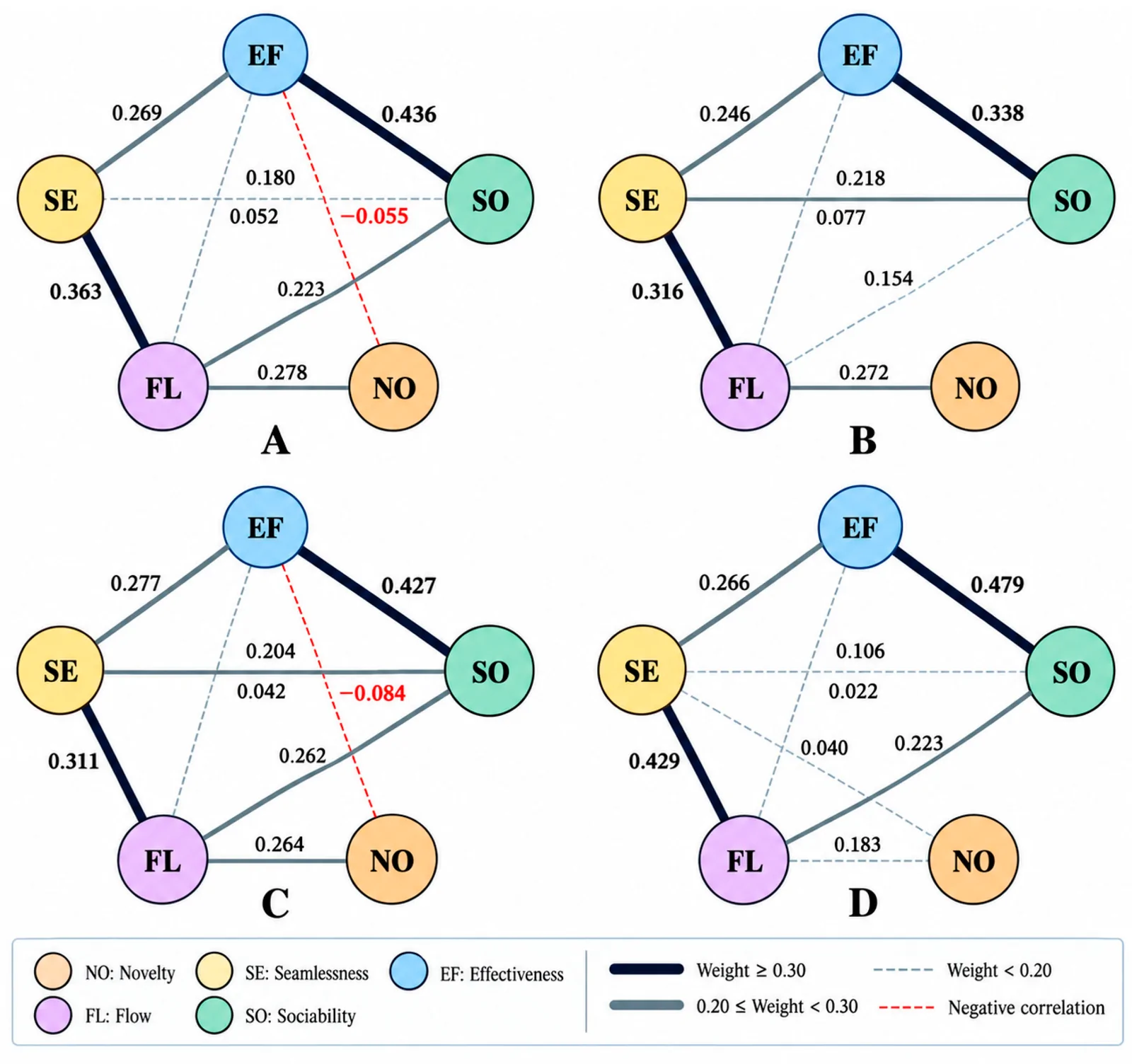
Structure of the estimated network. *Note:* (**A**) = total sample; (**B**) = L2 sample; (**C**) = Humanities & Social Sciences sample; (**D**) = STEM sample.

Using the EBICglasso estimation method (Epskamp et al., 2018), the AI-L2-ILES network was estimated with five nodes, including *Effectiveness*, *Sociability*, *Novelty*, *Flow*, and *Seamlessness*, with a theoretical maximum of 10 edges (see Figure 3). A total of eight non-zero edges were retained, resulting in a network density of 0.800, indicating a highly interconnected network structure.

The strongest edge was observed between *Sociability* and *Effectiveness* (SO–EF, 0.436, 95% CI [0.380, 0.493]), followed by *Seamlessness* and *Flow* (SE–FL, 0.363, 95% CI [0.293, 0.433]), *Novelty* and *Flow* (NO–FL, 0.278, 95% CI [0.216, 0.339]), *Seamlessness* and *Effectiveness* (SE–EF, 0.269, 95% CI [0.207, 0.332]), *Sociability* and *Flow* (SO–FL, 0.223, 95% CI [0.158, 0.288]), and *Sociability* and *Seamlessness* (SO–SE, 0.180, 95% CI [0.105, 0.255]). These findings suggest that learners’ perceptions of social interaction, learning effectiveness, and interaction continuity are closely interconnected within the AI-L2-ILES network.

In contrast, the edges between *Flow* and *Effectiveness* (FL–EF, 0.052, 95% CI [−0.007, 0.111]) and *Novelty* and *Effectiveness* (NO–EF, −0.055, 95% CI [−0.125, 0.015]) had confidence intervals including zero, indicating that these associations were not statistically reliable. Moreover, *Novelty* showed no direct connections with *Sociability* (NO–SO) or Seamlessness (NO–SE) after regularization, suggesting that *Novelty* represents a relatively distinct experiential dimension within the AI-L2-ILES network.

#### 4.2.2. Accuracy of Edge Weight Estimates

**Figure 4:**
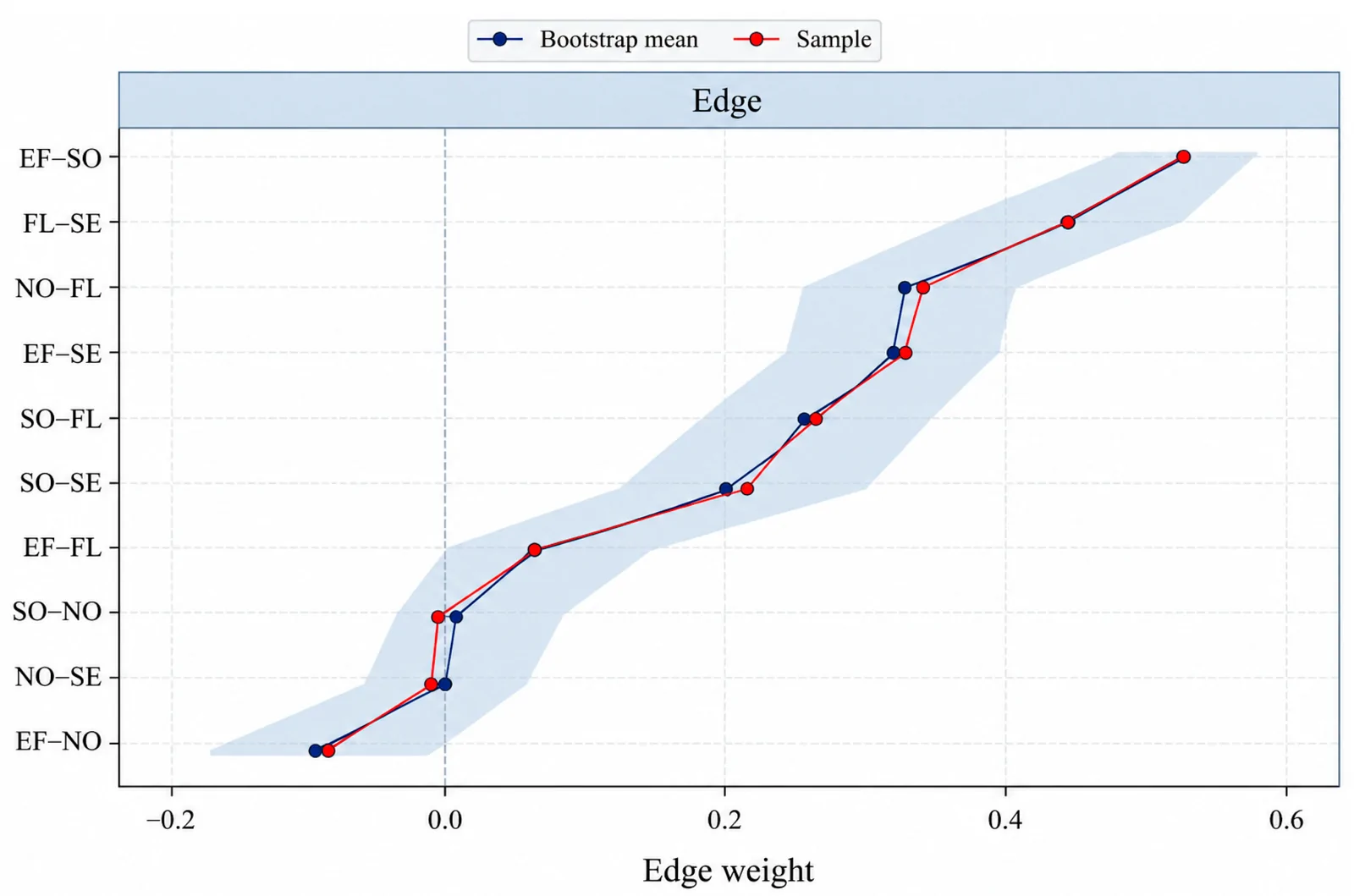
Accuracy of the estimated network. *Note*. EF = Effectiveness; SO = Sociability; NO = Novelty; FL = Flow; SE = Seamlessness.

Figure 4 illustrates the bootstrapped accuracy of the estimated edge weights. Based on 1000 non-parametric bootstrap iterations, six edges showed relatively narrow confidence intervals that did not include zero (SO–EF: 95% CI [0.380, 0.493]; SE–FL: [0.293, 0.433]; NO–FL: [0.216, 0.339]; SE–EF: [0.207, 0.332]; SO–FL: [0.158, 0.288]; SO–SE: [0.105, 0.255]), indicating that these edges were estimated with greater precision. The remaining four edges had confidence intervals that included zero (FL–EF: [−0.007, 0.111]; NO–EF: [−0.125, 0.015]; SO–NO: [−0.044, 0.044]; NO–SE: [−0.037, 0.037]), indicating less precise estimates.

Three types of edges should be distinguished in interpreting the network: edges regularized to zero (NO–SO, NO–SE), edges with non-zero weights but wide confidence intervals (none in the present network), and edges with non-zero weights and relatively narrow confidence intervals (e.g., SO–EF, SE–FL). The bootstrap confidence intervals primarily reflect estimation accuracy rather than serving as conventional significance tests for individual edges (Epskamp et al., 2018). The close correspondence between the original edge estimates and the bootstrap estimates (red and black lines, respectively) further supports the stability of the edge estimates.

#### 4.2.3. Centrality Analysis

**Figure 5:**
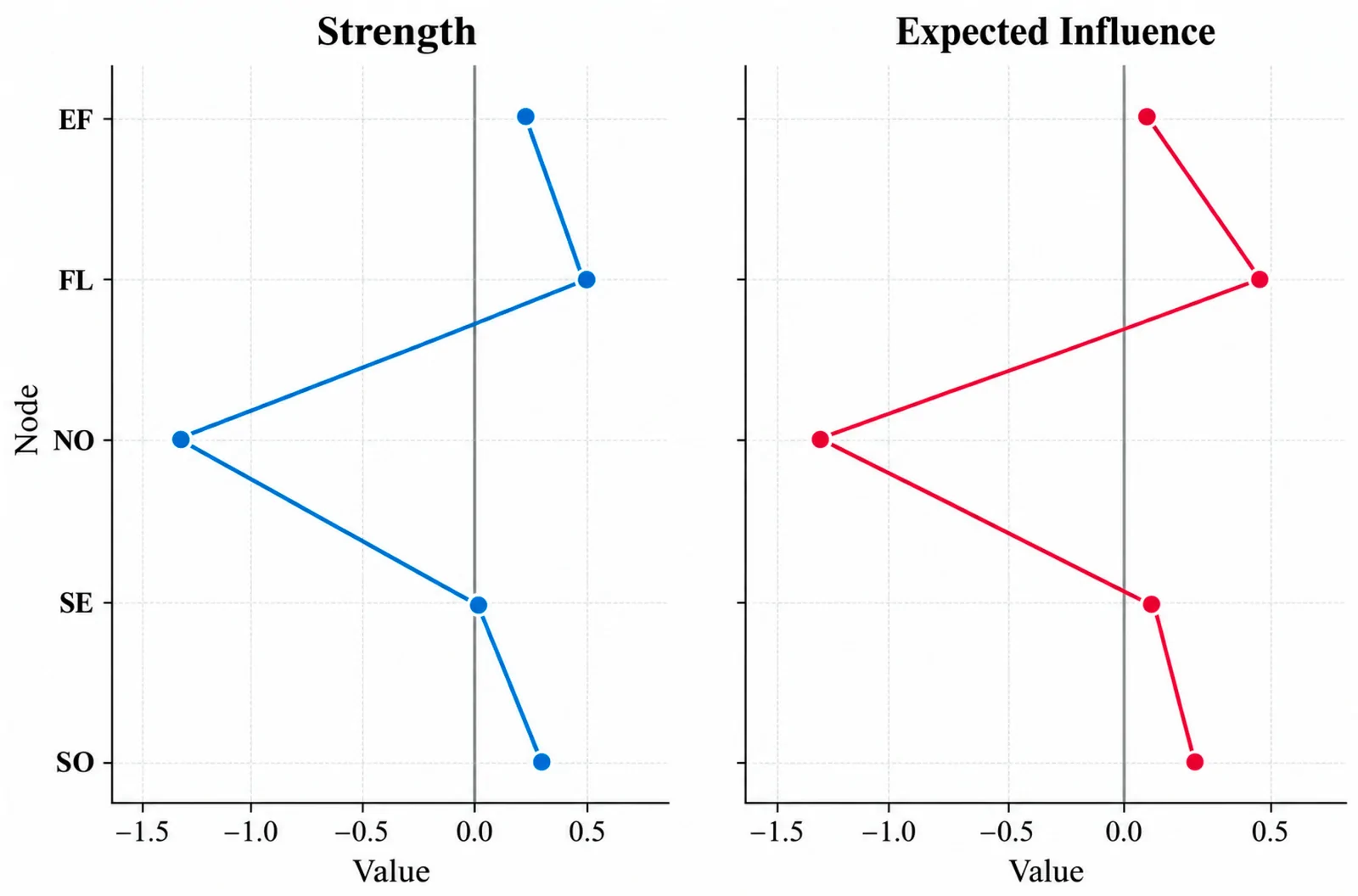
Centrality plot. *Note*. EF = Effectiveness; SO = Sociability; NO = Novelty; FL = Flow; SE = Seamlessness.

Centrality analysis was conducted to identify the relative importance of nodes within the AI-L2-ILES network (see Figure 5). Among the five dimensions, *Flow* demonstrated the highest strength centrality, indicating that it is the most strongly connected node within the network. In contrast, *Novelty* exhibited the lowest strength centrality, suggesting its relatively peripheral position. These findings highlight the central role of flow experience in connecting different dimensions of GenAI-mediated learning experiences.

#### 4.2.4. Centrality Stability

**Figure 6:**
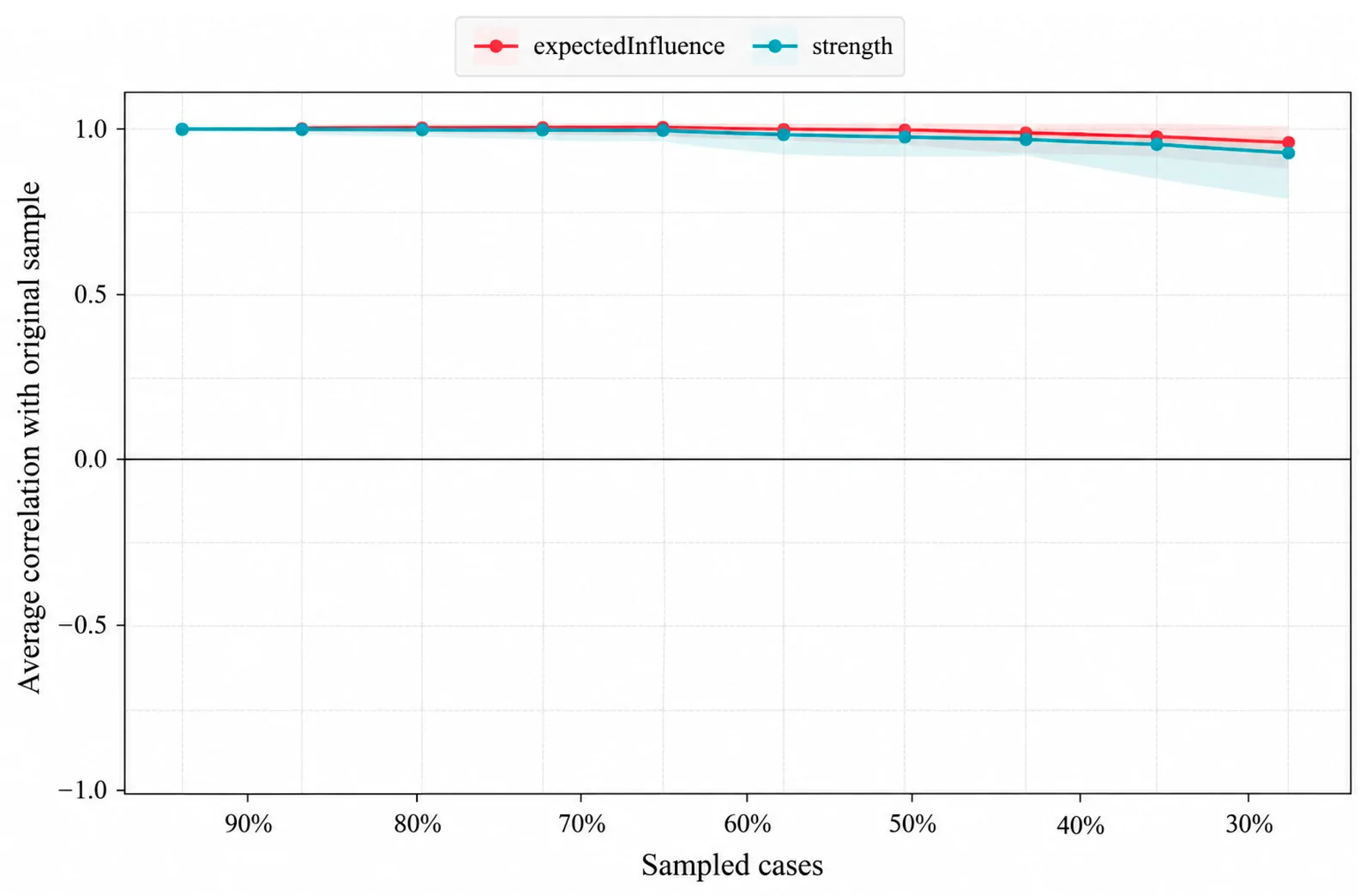
Centrality stability plot.

To assess the stability of the network estimates, we conducted a case-dropping bootstrap analysis with 1000 iterations (Epskamp et al., 2018). The CS coefficients for node strength and expected influence were both 0.75, exceeding the recommended threshold of 0.50 (see Figure 6), indicating good stability of the centrality estimates.

#### 4.2.5. Cross-Disciplinary Network Comparison

Pairwise Network Comparison Tests were conducted to examine whether the AI-L2-ILES network differed across academic disciplines (L2, Humanities and Social Sciences, and STEM). No significant differences were observed in global network strength among the three groups (L2 = 1.620, Humanities and Social Sciences = 1.873, STEM = 1.748).

Pairwise comparisons further showed no significant differences: L2 vs. Humanities and Social Sciences (S = 0.252, *p* = 0.249, FDR-corrected *p* = 0.522), L2 vs. STEM (S = 0.128, *p* = 0.348, FDR-corrected *p* = 0.522), and Humanities and Social Sciences vs. STEM (S = 0.125, *p* = 0.522, FDR-corrected *p* = 0.522). Similarly, the M-tests revealed no significant differences in network structure across groups (all FDR-corrected *p* > 0.736). These findings suggest that the AI-L2-ILES network structure remained stable across academic disciplines.

## 5. Discussion

In recent years, the application of GenAI in L2 education has expanded rapidly, moving beyond the provision of linguistic resources to supporting interactive learning processes. Previous studies have documented a range of functions served by GenAI in language education, including feedback provision, cognitive stimulation, and interaction facilitation (Lee et al., 2026). However, despite the increasingly prominent role of GenAI as an agent-like interactive partner rather than merely a learning tool, reliable instruments specifically designed to capture L2 learners’ cognitive evaluation of their interactive learning experiences with GenAI remain scarce. To address this research gap, the present study developed the AI-L2-ILES and evaluated its psychometric properties.

### 5.1. Psychometric Properties of the AI-L2-ILES

Regarding the factor structure examined in ***RQ1a***, the eight dimensions of the original SSIE framework (Zhang et al., 2025) underwent a process of retention, deletion, reconceptualization, and restructuring during contextual adaptation, resulting in a five-dimensional model consisting of *Effectiveness*, *Sociability*, *Novelty*, *Flow*, and *Seamlessness*. The removal of *Ease of Use* and *Safety* suggests that, in GenAI-assisted L2 learning contexts, learners’ evaluations are less focused on technical attributes such as system usability and privacy protection, but rather on whether GenAI-mediated interactions can effectively facilitate L2 learning. This finding aligns with Lee et al. (2026), who emphasized that the central value of GenAI in L2 education lies in its role as a cognitive stimulator and interaction facilitator. Although *Safety* represents important concerns in traditional smart service contexts, such as payment security and personal information protection, these concerns may be less salient in learners’ routine use of GenAI for L2 learning, providing contextual justification for its exclusion. Notably, P. Li (2026), who applied the SSIE framework to a GenAI-assisted music education context, also found that *Safety* negatively predicted learners’ behavioral intentions. This finding further supports our study’s decision to exclude *Safety* and suggests that, in GenAI-supported educational contexts, safety may not function as a core experiential dimension and may, under certain conditions, even attenuate learners’ positive perceptions.

For the dimension restructuring examined in ***RQ1a***, the original SSIE dimensions of *Efficiency* and *Learning* (Zhang et al., 2025) did not emerge as distinct factors. Instead, three *Efficiency* items converged with one retained *Learning* item to form a single factor, which was subsequently conceptualized as *Effectiveness*. Although this factor was identified inductively, its emergence can be theoretically interpreted in light of the goal-oriented nature of GenAI-assisted L2 learning. In service contexts, *Efficiency* primarily concerns convenience and reduced effort, whereas *Learning* concerns knowledge acquisition. In educational contexts, however, the value of efficient interactions may depend partly on whether they contribute to meaningful learning outcomes. Consistent with this view, Suzuki et al. (2025) found that learning efficiency during computer-assisted L2 vocabulary learning strongly predicted subsequent vocabulary retention. This does not suggest that efficiency and learning are psychometrically identical; rather, it indicates a functional relationship between the resources required for learning and the outcomes achieved. Accordingly, the convergence observed here may reflect learners’ holistic evaluations of whether GenAI interactions enable meaningful L2 learning with a reasonable investment of time and effort. Nevertheless, the observed convergence may also have been influenced by the limited number of retained *Learning* items, and should therefore be interpreted cautiously rather than as evidence that Efficiency and Learning are inherently identical constructs. Regarding ***RQ1b*** and ***RQ1c***, the AI-L2-ILES demonstrated satisfactory reliability, validity, and measurement invariance, indicating sound psychometric properties (Cheung & Rensvold, 2002; Fornell & Larcker, 1981).

The retention and reconceptualization of *Sociability* represent an important finding supporting the construct validity of the scale. In the original SSIE framework, *Socialization* primarily refers to interpersonal emotional connections among users in service contexts (Zhang et al., 2025). In this study, however, this dimension was reconceptualized as learners’ perceptions of social responsiveness and partner-like communication during interactions with GenAI. The CFA results further supported the validity of this reconceptualized dimension, as indicated by its satisfactory factor loadings. This finding aligns with the Computers Are Social Actors paradigm proposed by Reeves and Nass (1996) and Nass and Moon (2000), which suggests that individuals naturally apply social rules and expectations to technological systems during interactions. Such social responses may be particularly salient in L2 learning contexts, where language learning is inherently social. The immediate dialogue, personalized feedback, and natural language interaction enabled by GenAI may further enhance learners’ perceptions of GenAI as a social actor (Luo & Gan, 2025). This finding is also consistent with the development of the Personal Attributes for Self-Directed AI Learning scale, which showed that learners tend to perceive GenAI as a responsive learning partner rather than merely a technological tool (B. Li et al., 2025). Therefore, *Sociability* not only maintains theoretical continuity with prior smart interaction research but also acquires new context-specific meanings within GenAI-assisted L2 learning.

### 5.2. Dimensional Interconnection of the AI-L2-ILES

Concerning the interrelationships among dimensions examined in **RQ2**, *Sociability* demonstrated the strongest association with *Effectiveness*, suggesting that learners perceiving greater social responsiveness during GenAI interactions tended to evaluate these interactions as more effective for learning. L2 learning is inherently socially mediated, with learners developing language abilities through communication, feedback, and negotiation of meaning (Lee et al., 2026). When GenAI provides partner-like responsiveness and conversational support, learners may be more likely to perceive such interactions as beneficial. F. Wang et al. (2025) suggested that perceived GenAI interactivity shares psychological continuity with traditional interpersonal interactions and may support GenAI-supported learning processes. Thus, enhanced social responsiveness may be associated with more positive evaluations of GenAI-mediated learning experiences. The strong association between *Sociability* and *Effectiveness* also supports the reconceptualization of *Sociability* in the present scale, as excluding this dimension would overlook an important aspect of AI-L2-ILE. Moreover, the strong association between *Seamlessness* and *Flow* indicates that smooth and uninterrupted GenAI interactions may be linked to learners’ concentration and immersion. When interactions are not disrupted by technical delays, incomplete responses, or loss of conversational context, learners may be more likely to achieve a focused learning state.

*Novelty* demonstrated the most independent position within the network, showing relatively weak associations with the other dimensions. Its connections with *Sociability* and *Seamlessness* were further reduced to near zero after regularization. This finding suggests that learners’ perceptions of GenAI novelty are relatively distinct from their evaluations of social, fluent, and immersive interaction experiences. *Novelty* mainly reflects learners’ initial cognitive responses to GenAI as an innovative technology, whereas other dimensions capture deeper experiences developed through sustained interactions. Several alternative explanations for *Novelty*’s peripheral position should be considered. First, this pattern may reflect the sample’s relatively high GenAI familiarity, as participants were recruited based on prior GenAI use; for experienced users, novelty may be less salient. Second, novelty effects tend to diminish over time, and the cross-sectional design captures only a single time point, potentially obscuring novelty’s role during initial adoption. Third, the novelty items focused specifically on perceptions of newness, which may be closer to initial impressions than sustained experiential appraisals. These explanations are not mutually exclusive, and future longitudinal research could clarify whether *Novelty*’s peripheral position is a stable structural feature or reflects user experience and measurement timing. Overall, the findings suggest that although *Novelty* may encourage initial engagement, it is unlikely to be a core dimension of sustained GenAI-mediated learning experience.

In contrast, *Flow* emerged as the most central dimension, ranking highest across multiple centrality indices, suggesting that it may function as a statistical bridge connecting other dimensions within the network. As an immersive state characterized by focused attention and sustained involvement, *Flow* may relate to learners’ evaluations of *Effectiveness, Sociability*, and *Seamlessness*. Given the cross-sectional nature of the data, however, these findings reflect associations rather than causal priority. The centrality of *Flow* carries both theoretical and measurement-related implications. Theoretically, *Flow*’s hub-like position suggests that immersive engagement may function not merely as one of several parallel experiential facets, but as a state that connects with and potentially reinforces other dimensions of the interaction experience. This interpretation is consistent with prior research conceptualizing flow as a state of optimal engagement in L2 learning (Csikszentmihalyi, 1990; Jiang & Li, 2026), as well as evidence linking flow with satisfaction and perceived learning in technology-enhanced language learning (H. Liu & Song, 2021). The present findings extend this literature by demonstrating *Flow*’s network-level prominence in GenAI-assisted L2 interaction. From a measurement perspective, *Flow*’s central role does not imply that other dimensions are less important or that the AI-L2-ILES should be reduced to a unidimensional measure. Rather, if researchers seek a brief screener of learners’ overall interaction experience, *Flow*-related items may provide efficient indicators, whereas the full five-dimensional structure remains important for comprehensive diagnostic assessment.

Notably, the network comparison analysis did not detect significant structural differences across learners from different academic disciplines. This finding complements previous research documenting disciplinary variations in GenAI use and L2 learning (Xie, 2019; Y. Xu & Wu, 2026) by suggesting that, in terms of how learners evaluate their GenAI interaction experiences, network structures may be more similar than different across disciplines. However, the absence of detected differences should not be taken as strong evidence of full structural equivalence. It may reflect the broad discipline categories used in this study, limited sensitivity of the Network Comparison Tests, or relatively similar GenAI exposure across the sample. Moreover, because the findings are based on self-report scale data, they do not directly capture actual interaction processes, behavioral patterns, or language outcomes. Thus, the observed similarity should be interpreted primarily as a pattern in learners’ self-reported evaluations rather than evidence of the underlying cognitive architecture of human–GenAI interaction. One possibility is that disciplinary differences may relate to why learners use GenAI more than how they evaluate the interaction experience itself, but this interpretation remains tentative and requires further investigation.

## 6. Implications and Limitations

This study makes three theoretical contributions. First, it proposes and validates the construct of AI-L2-ILE, conceptualized as a comprehensive experiential evaluation formed by L2 learners through their perceptions of the interaction process, interactional relationship, and learning value during sustained interactions with GenAI. This construct expands the conceptual boundaries of GenAI-assisted language learning research by shifting the focus from the functional attributes of GenAI to the structure of learners’ experiential evaluations, offering a learner-centered perspective for understanding how GenAI is experienced as an interactive learning partner in L2 learning. Second, this study identifies and validates the five-factor structure of the AI-L2-ILES (*Effectiveness*, *Sociability*, *Novelty*, *Flow*, and *Seamlessness*). The emergence of *Effectiveness* demonstrates how the goal-oriented nature of educational contexts can shape experiential dimensions: when learners evaluate GenAI-mediated interaction in relation to their learning goals, experiential quality may become closely linked to perceived learning value. This structural insight informs both L2 research and AI-in-education studies by specifying how learners distinguish different aspects of their interactions with GenAI in L2 learning. Third, this study introduces dimension-level network analysis into research on AI-L2-ILE and reveals differentiated association patterns among dimensions, including the strong connection between *Sociability* and *Effectiveness*, the relative independence of *Novelty*, and the central role of *Flow*. These findings provide a complementary perspective beyond traditional dimensional approaches for understanding the internal structure of this construct, with implications for understanding how learners experience GenAI through its interactive qualities in L2 learning.

This study also offers several practical implications for GenAI-assisted L2 teaching. First, the AI-L2-ILES serves as a diagnostic instrument for educators to identify potential weaknesses in learners’ GenAI interaction experiences. By assessing learners’ experiences across dimensions such as *Effectiveness* and *Flow*, teachers can develop more targeted instructional strategies and optimize GenAI integration in language learning. Second, the strong association between *Sociability* and *Effectiveness* suggests that learners who perceive greater social responsiveness from GenAI also tend to report higher perceptions of its learning value. Therefore, teachers and GenAI developers should attend not only to information accuracy but also to interactional qualities, including responsive feedback, empathetic support, and coherent dialogue. Third, the central role of *Flow* highlights the importance of immersive experiences in connecting different aspects of GenAI interaction. Instructional designers should thus prioritize interaction fluency, task engagement, and conversational continuity while minimizing technical disruptions and cognitive distractions. Finally, the peripheral position of *Novelty* indicates that although *Novelty* may be associated with initial adoption, sustained engagement should be supported by meaningful learning experiences rather than temporary technological appeal. These implications also inform the use and design of GenAI for L2 learning by suggesting that social responsiveness and flow-sustaining features may deserve greater attention than novelty-driven functionalities.

Several limitations should be acknowledged. First, the sample was limited to Chinese university students recruited through convenience sampling, which restricts generalizability to other cultural, educational, or age groups. Learners from different contexts, such as secondary school students, adult learners, or non-academic populations, may develop different evaluations of GenAI-mediated learning experience. Second, the study relied entirely on self-report data collected from the same participants at a single time point. This raises concerns about common-method variance and social desirability biases, which may have inflated the observed correlations, including the criterion-related validity coefficient with GenAI-IDLE. Third, the cross-sectional design precludes causal inferences. Although network analysis revealed associations among dimensions, these reflect statistical relationships rather than causal priority. For example, *Flow*’s centrality does not establish that enhancing *Flow* would causally improve perceptions of *Effectiveness* or *Sociability*. Fourth, the study did not include objective behavioral data (e.g., actual GenAI usage logs, task completion records, or interaction frequency) to complement the self-report findings. Such data would help validate whether the AI-L2-ILES scores correspond to observable interaction patterns. Fifth, this study treated GenAI as a general category and did not differentiate among specific tools such as ChatGPT, DeepSeek, or Kimi, which may afford different interaction experiences due to variations in interface design, response style, and functional affordances. Future research could address these limitations through more diverse samples, longitudinal or experimental designs, objective usage data, and comparative studies across GenAI platforms.

## 7. Conclusions

This study developed and validated the AI-L2-ILES, a five-dimensional instrument comprising *Effectiveness*, *Sociability*, *Novelty*, *Flow*, and *Seamlessness*. The scale demonstrated strong psychometric properties, including satisfactory reliability, validity, and measurement invariance across genders and academic disciplines. Dimension-level network analysis further uncovered the structural relationships among these dimensions. The strong association between *Sociability* and *Effectiveness* underscores the critical role of social responsiveness in shaping L2 learners’ GenAI-mediated interactive learning experiences, whereas the highest centrality of *Flow* indicates that immersive experience functions as a central component linking multiple experiential dimensions. In contrast, the relative independence of *Novelty* highlights the distinction between initial perceptions of technological novelty and deeper, sustained experiential evaluations. Overall, the AI-L2-ILES appears to be a promising instrument for future research, though further validation is needed across diverse contexts, including different countries, L2s, learner proficiency levels, specific GenAI tools, and varied learning tasks, before its generalizability can be fully established.

## Figures and Tables

**Table 1:** Demographic details of participants.

Variables	EFA	CFA
Gender		
Male	211 (37.3%)	292 (51.7%)
Female	354 (62.7%)	273 (48.3%)
Age		
≤20	293 (51.9%)	279 (49.4%)
21–25	229 (40.5%)	195 (34.5%)
26–30	31 (5.5%)	69 (12.2%)
>30	12 (2.1%)	22 (3.9%)
Major		
L2	187 (33.1%)	146 (25.8%)
H & SS	232 (41.1%)	214 (37.9%)
STEM	146 (25.8%)	205 (36.3%)
Grade		
Undergraduate	296 (52.4%)	280 (49.6%)
Master student	214 (37.9%)	177 (31.3%)
Ph.D. student	55 (9.7%)	108 (19.1%)

**Table 2:** Examples of item adaptation from SSIE to AI-L2-ILES.

SSIE Original Item	Adapted Item for AI-L2-ILES	Adaptation Strategy
Smart technologies made my family’s stay at the hotel more efficient.	GenAI makes my L2 learning more efficient.	Domain substitution: “smart technologies” → “GenAI”; “hotel stay” → “L2 learning”
Smart technologies improved my family’s social experience at the hotel.	Using GenAI for L2 learning feels like interacting with a real person.	Conceptual redefinition: social experience → social responsiveness
When using smart technologies, I was totally absorbed in what I was doing.	Using GenAI for L2 practice makes me fully absorbed.	Domain substitution: “smart technologies” → “GenAI”; “what I was doing” → “L2 practice”

**Table 3:** EFA results of the AI-L2-ILES.

Factor	SS Loadings	Items	Loadings					Communalities	Variance Explained
Effectiveness	2.495	EF1	**0.788**	−0.010	−0.007	−0.051	−0.003	0.586	15.6%
		EF2	**0.951**	0.036	−0.150	0.050	−0.027	0.755	
		EF3	**0.780**	0.004	0.057	−0.046	0.033	0.670	
		EF4	**0.540**	−0.055	0.249	0.103	0.029	0.622	
Sociability	2.381	SO1	0.039	**0.878**	0.030	−0.038	−0.017	0.751	14.9%
		SO2	−0.030	**0.991**	0.031	−0.026	0.024	0.981	
		SO3	−0.007	**0.776**	−0.034	0.077	0.005	0.645	
Novelty	2.083	NO1	−0.027	0.077	**0.805**	0.029	−0.073	0.696	13.0%
		NO2	−0.120	−0.016	**0.854**	0.029	−0.012	0.624	
		NO3	0.067	0.059	**0.736**	−0.072	0.083	0.645	
Flow	1.926	FL1	−0.027	0.077	0.008	**0.804**	−0.049	0.644	12.0%
		FL2	−0.050	−0.057	0.064	**0.838**	0.082	0.786	
		FL3	0.057	−0.008	−0.043	**0.690**	−0.002	0.471	
Seamlessness	1.908	SE1	0.001	0.025	−0.072	0.102	**0.722**	0.565	11.9%
		SE2	0.041	0.013	−0.013	−0.113	**0.884**	0.702	
		SE3	−0.034	−0.036	0.094	0.068	**0.730**	0.650	

*Note.* Extraction method = maximum likelihood; rotation method = Promax. Bold values indicate the primary factor loadings. All loadings are reported to three decimal places, including cross-loadings. The five factors accounted for 67.5% of the total variance.

**Table 4:** Factor correlations for the final five-factor solution.

Factor	1	2	3	4	5
1. Effectiveness	—				
2. Sociability	0.627 ***	—			
3. Novelty	0.007	0.112 **	—		
4. Flow	0.356 ***	0.500 ***	0.360 ***	—	
5. Seamlessness	0.526 ***	0.585 ***	0.181 ***	0.599 ***	—

*Note*. ** *p* < 0.01, *** *p* < 0.001.

**Table 5:** Definition of the five dimensions.

Dimensions	Definition
Effectiveness	Perceived goal attainment and learning benefits derived from GenAI interaction in L2 learning.
Sociability	Perceived social responsiveness and the partner-like qualities of GenAI interaction in L2 learning.
Novelty	Perceived newness and innovativeness of the GenAI-assisted L2 learning experience.
Flow	Perceived immersion, absorption, and focused engagement during GenAI interaction in L2 learning.
Seamlessness	Perceived smoothness and continuity of GenAI-mediated L2 interactions.

**Table 6:** CFA results for the AI-L2-ILES.

Dimension	Item	B	β	*p*	S.E.	C.R.	Residual Variance
Effectiveness	EF1	1.000	0.766	—	—	—	0.340
	EF2	1.053	0.845	<0.001	0.051	20.630	0.215
	EF3	1.134	0.830	<0.001	0.057	19.879	0.282
	EF4	0.949	0.744	<0.001	0.054	17.695	0.352
Sociability	SO1	1.000	0.835	—	—	—	0.303
	SO2	1.025	0.816	<0.001	0.047	21.805	0.369
	SO3	0.950	0.836	<0.001	0.043	22.173	0.271
Novelty	NO1	1.000	0.870	—	—	—	0.437
	NO2	1.141	0.941	<0.001	0.041	27.496	0.229
	NO3	0.969	0.753	<0.001	0.045	21.529	0.976
Flow	FL1	1.000	0.798	—	—	—	0.438
	FL2	1.039	0.872	<0.001	0.048	21.777	0.261
	FL3	0.927	0.724	<0.001	0.053	17.399	0.597
Seamlessness	SE1	1.000	0.830	—	—	—	0.343
	SE2	0.983	0.813	<0.001	0.045	21.622	0.376
	SE3	0.967	0.827	<0.001	0.046	21.007	0.328

*Note*. B = unstandardized factor loading; β = standardized factor loading; S.E. = standard error; C.R. = critical ratio. Residual variance refers to the unstandardized variance of the item-specific error term. The unstandardized loading of the first item for each factor was fixed at 1.000 for model identification.

**Table 7:** Factor covariances of the five-factor CFA model.

Factor Pair	Covariance	*p*
Effectiveness ↔ Sociability	0.431	<0.001
Effectiveness ↔ Novelty	0.081	0.034
Effectiveness ↔ Flow	0.338	<0.001
Effectiveness ↔ Seamlessness	0.409	<0.001
Sociability ↔ Novelty	0.163	<0.001
Sociability ↔ Flow	0.467	<0.001
Sociability ↔ Seamlessness	0.471	<0.001
Novelty ↔ Flow	0.371	<0.001
Novelty ↔ Seamlessness	0.165	<0.001
Flow ↔ Seamlessness	0.538	<0.001

**Table 8:** Model fit comparison for the AI-L2-ILES.

Model	χ^2^	*df*	χ^2^/*df*	CFI	TLI	RMSEA	SRMR
One-factor	2269.148	104	21.819	0.615	0.556	0.192	0.133
Four-factor	558.340	98	5.697	0.918	0.900	0.091	0.048
Five-factor	287.850	94	3.062	0.966	0.956	0.060	0.039
Higher-order	365.957	99	3.697	0.953	0.942	0.069	0.061

**Table 9:** Discriminant and convergent validity of the AI-L2-ILES.

Dimensions	CR	AVE	1	2	3	4	5
1. Effectiveness	0.874	0.635	**0.797**				
2. Sociability	0.868	0.687	0.744 ***	**0.829**			
3. Novelty	0.893	0.737	0.100 *	0.167 ***	**0.858**		
4. Flow	0.842	0.641	0.555 ***	0.639 ***	0.363 ***	**0.800**	
5. Seamlessness	0.863	0.678	0.676 ***	0.648 ***	0.162 ***	0.705 ***	**0.823**

*Note*: CR = Composite Reliability, AVE = Average Variance Extracted, * *p* < 0.05; *** *p* < 0.001; Bold values indicate the square roots of AVE.

**Table 10:** HTMT analysis.

Dimensions	1	2	3	4	5
1. Effectiveness	—				
2. Sociability	0.765	—			
3. Novelty	0.103	0.168	—		
4. Flow	0.581	0.654	0.380	—	
5. Seamlessness	0.685	0.646	0.164	0.715	—

**Table 11:** Measurement invariance across genders and academic disciplines.

Model	χ^2^	*df*	χ^2^/*df*	TLI	CFI	RMSEA	SRMR	ΔCFI	ΔRMSEA	Evaluation
Gender										
M1	419.734	188	2.233	0.948	0.959	0.047	0.048	-	-	Supported
M2	427.179	199	2.147	0.951	0.960	0.045	0.049	0.001	−0.002	
M3	456.079	215	2.121	0.953	0.957	0.045	0.049	−0.003	0.000	
Discipline										
M1	592.812	282	2.102	0.932	0.946	0.047	0.057	-	-	Supported
M2	625.203	304	2.057	0.934	0.945	0.045	0.058	−0.001	−0.002	
M3	664.280	336	1.977	0.939	0.943	0.045	0.058	−0.002	0.000	

*Note*: M1 = configural model; M2 = metric model; M3 = scalar model.

## Data Availability

The data supporting the findings of this study were collected as part of an ongoing nationally funded research project and constitute one of the project’s interim research outputs. Because the project is still in progress, the dataset is not currently available for unrestricted public access. However, the data can be obtained from the corresponding author upon reasonable request.

## Supplementary Materials

The following supporting information can be downloaded at: https://www.mdpi.com/article/10.3390/jintelligence14090226/s1.

## Abbreviations

The following abbreviations are used in this manuscript:

GenAI	Generative artificial intelligence
L2	Second language
AI-L2-ILE	GenAI L2 Interactive Learning Experience
AI-L2-ILES	GenAI L2 Interactive Learning Experience Scale
SSIE	Smart Service Interactive Experience
EFA	Exploratory factor analysis
CFA	Confirmatory factor analysis
IDLE	Informal digital learning of English
