# Supplementary material for: Second Language Learners’ Cognitive Evaluation of GenAI Interactive Learning Experience: Scale Development and Network Analysis
Source: J Intell. 2026 Sep 19;14(9):226. doi: 10.3390/jintelligence14090226 (PMC13608319; doi:10.3390/jintelligence14090226)
Supplement: Supplementary file 1 [file jintelligence-14-00226-s001.zip › jintelligence-4515350-supplementary.pdf]

Supplementary Materials

The GenAI L2 Interactive Learning Experience Scale (AI-L2-ILES)

Administration Instructions

This questionnaire is designed to understand your interactive learning experiences with generative artificial intelligence (GenAI) tools (e.g., ChatGPT, DeepSeek, Kimi) in L2 learning. Please read each statement carefully and indicate the extent to which you agree or disagree based on your actual experiences using GenAI for L2 learning. There are no right or wrong answers. Please respond to all items.

Response Anchors

Please indicate your level of agreement with each statement using the following 6-point Likert scale:

1 = Strongly Disagree
2 = Disagree
3 = Somewhat Disagree
4 = Somewhat Agree
5 = Agree
6 = Strongly Agree

Dimension	No	English item	Chinese item
Effectiveness	1	GenAI makes my L2 learning more efficient.	GenAI 能够提高我的二语学习效率。
	2	GenAI helps me quickly obtain customized L2 learning materials.	GenAI 能够帮助我快速获得符合自身需求的二语学习材料。
	3	GenAI provides support for my L2 learning with little effort.	GenAI 能够轻松地支持我的二语学习。
	4	GenAI's personalized support enhances my L2 learning.	GenAI 提供的个性化支持能够提升我的二语学习效果。
Sociability	5	Using GenAI for L2 learning feels like interacting with a real person.	使用 GenAI 进行二语学习时，我感觉自己像是在与真人交流。
	6	GenAI's responses make me feel understood in my L2 learning.	在二语学习过程中，GenAI 的回应让我感到它理解我的需求。

	7	GenAI creates a sense of social interaction during my L2 learning.	在二语学习过程中，与 GenAI 互动让我感受到一种类似人与人之间交流的感觉。
Novelty	8	I perceive GenAI-assisted L2 learning as novel.	我认为借助 GenAI 进行二语学习是一种新颖的学习体验。
	9	GenAI introduces new ways of learning a L2.	GenAI 为二语学习带来了新的学习方式。
	10	GenAI creates distinctive L2 learning experiences.	GenAI 能够为我带来独特的二语学习体验。
Flow	11	Interacting with GenAI for L2 learning keeps me focused.	在二语学习过程中与 GenAI 互动能够让我保持专注。
	12	Using GenAI for L2 practice makes me fully absorbed.	使用 GenAI 进行二语练习时，我能够全身心地投入其中。
	13	Learning L2 with GenAI makes me lose track of time.	使用 GenAI 进行二语学习时，我常常会忘记时间。
Seamlessness	14	GenAI enables smooth L2 learning interactions.	GenAI 能够让二语学习互动更加顺畅。
	15	GenAI allows continuous interaction during my L2 learning.	在二语学习过程中，我可以持续与 GenAI 进行互动。
	16	GenAI supports an uninterrupted L2 learning process.	GenAI 能够让我保持连贯、不间断的二语学习过程。

Language Version

Chinese (original). All items were initially developed and administered in Chinese for Chinese-speaking participants. The English translation provided above is for reference purposes only and does not constitute a formally validated version. Researchers wishing to use the scale in other language contexts are advised to conduct appropriate linguistic validation procedures (e.g., translation and back-translation).

Scoring Procedure

All items are rated on a 6-point Likert scale ranging from 1 (*Strongly Disagree*) to 6 (*Strongly Agree*). No items are reverse-coded.

Subscale scores are calculated as the mean of the items within each dimension:

***Effectiveness*:** Items 1–4 (4 items)

***Sociability*:** Items 5–7 (3 items)

Novelty: Items 8–10 (3 items)

Flow: Items 11–13 (3 items)

Seamlessness: Items 14–16 (3 items)

Interpretation of Subscale Scores

**Dimensions**	**Definition**
Effectiveness	Perceived goal attainment and learning benefits derived from GenAI interaction in L2 learning.
Sociability	Perceived social responsiveness and the partner-like qualities of GenAI interaction in L2 learning.
Novelty	Perceived newness and innovativeness of the GenAI-assisted L2 learning experience.
Flow	Perceived immersion, absorption, and focused engagement during GenAI interaction in L2 learning.
Seamlessness	Perceived smoothness and continuity of GenAI-mediated L2 interactions.
